# Evaluation of burnout level in working people who decided to quit smoking

**DOI:** 10.1007/s11845-026-04329-8

**Published:** 2026-03-31

**Authors:** Asena Merve Oflaz, Hilal Aksoy, Izzet Fidanci, Ismail Arslan, Duygu Ayhan Başer

**Affiliations:** 1 Department of Family Medicine, Faculty of Medicine, Hacettepe University, Ankara, Türkiye; 2 Department of Family Medicine, Ankara Education and Research Hospital, Ankara, Türkiye

**Keywords:** Tobacco, Burnout, Smoking cessation, Addiction, Nicotine

## Abstract

**Background:**

Nicotine addiction is the most common and important type of substance addiction and has lots of harms to human health. The most important thing a smoker can do to protect their health is to quit smoking. When deciding to quit smoking, the relationships of a person with people he/she lives with also affect the quitting process positively or negatively.

**Aim:**

The purpose of this study was to define the couple-burnout and professional burnout experienced by individuals who were applying to smoking cessation clinics for the first time.

**Methods:**

The study was carried out at the Smoking Cessation Polyclinics. Participants were given a survey form that included questions about their sociodemographics and reasons for starting to smoke. Also “Modified Fagerstrom Test” “Burnout Scale short form,” and “Couple-burnout Scale-short form” were used. The statistical analysis was conducted using IBM SPSS Statistics for Windows, Version 23.

**Results:**

People who were unhappy with their jobs (*p* = 0.035), worked shifts (*p* = 0.038), or suffered from mental illnesses (*p* = 0.005) had higher levels of burnout. Women are more likely than males to experience couple burnout (*p* = 0.001) and to be dissatisfied with their careers (*p* = 0.031). A significant positive connection (*p* < 0.001) was found between the spouse burnout scale and the burnout scale.

**Conclusion:**

In people who decide to quit smoking, the underlying burnout should definitely be taken into consideration. Spousal support should also be considered among other factors during polyclinic visits.

**Supplementary Information:**

The online version contains supplementary material available at 10.1007/s11845-026-04329-8.

## Introduction

Nicotine addiction is the most common and important type of substance addiction [1]. Tobacco use is a major risk factor for cardiovascular disease, respiratory diseases, more than twenty types of cancer, and many other diseases [2]. The most important thing a smoker can do to protect their health is to quit smoking. When you quit smoking, the risk of developing smoking-related diseases will decrease, and if a disease is present, the rate of progression of that disease will slow down [1].

Beyond the physiological dependence, psychological and emotional factors such as burnout may play a crucial role in both maintaining and quitting smoking habits. The concept of burnout was first used by Freudenberger to describe the exhaustion observed in volunteer workers in medical field. Later, Maslach and Jackson defined burnout as a syndrome of physical, emotional and mental exhaustion that marked by physical exhaustion, chronic fatigue, feelings of helplessness and hopelessness, development of a negative self-concept, and negative attitudes towards profession, life in general, and other people [3].

While burnout syndrome was initially thought to be a condition that only occurs in certain occupational groups, the studies conducted in later years have shown that burnout is not a condition that only occurs in people working in occupations with face-to-face interaction but also can occur in all occupational groups [4, 5]. It is stated that in a case of burnout, behavioral symptoms include postponing or procrastinating on certain things. For this reason, people may postpone their plans to quit smoking and may not want to quit at all. However, since the emotional exhaustion experienced in a case of burnout will lead to a decrease in a person’s desires, the desire to smoke may also decrease and therefore they may consider to quit smoking.

In the study conducted by Velioglu et al. in 2019 with 2119 participants who applied to the Smoking Cessation Polyclinic, it was determined that as nicotine addiction increased, depression and burnout levels also increased significantly [6]. In a review conducted by Yargic and Baykan in 2013, it was stated that the rate of adults who are addicted to nicotine having suffered from depression in the last year is twice as high as that of those who are not addicted to nicotine [7].

According to Pines, people feel the need to believe that life is meaningful and tend to seek what is meaningful to them. They also tend to replace their unfulfilled expectations and dreams of their childhood with meaningful childhood experiences. In this sense, people want to choose a job and a spouse that will meet their unfulfilled childhood needs and give meaning to their childhood experiences. However, when people fail in these choices, they re-experience similar traumas and burnout occurs [8–13]. Professional burnout and spousal burnout affect each other. When an individual with professional burnout is looked into, it may be discovered that the real problem is their marriage or relationship, or vice versa [10].

When we searched the literature, we came across studies comparing depression and anxiety levels in people who decided to quit smoking, using scales. However, we could not find any studies measuring burnout, which is a more advanced state of emotional depression. In addition, when deciding to quit smoking, the relationships of a person with people he/she lives with also affect the quitting process positively or negatively. Therefore, in our study, we aimed to define the burnout states [both occupational and spouse-related] of individuals who applied to the smoking cessation clinics in order to better understand the emotional aspects of individuals who decided to quit smoking and to determine the smoking cessation method more accurately.

## Materials and methods

### Ethics

For this research, the permission was received from ……University Non-Interventional Clinical Research Ethics Committee with the decision number 2022/13–62 dated 06.09.2022.

### Design

The research is descriptive in nature. The universe of the research consists of people between the ages of 18–65 who applied for their first examination to the …….Faculty of Medicine, Department of Family Medicine, Smoking Cessation Polyclinic and ……….Training and Research Hospital, Family Medicine Clinic, Smoking Cessation Polyclinic. The research was conducted between November 1st 2022 and May 1st 2023.

The average monthly visit to the two polyclinics is 100, of which approximately 50 are new applicants. Data was collected over six months, and it had beenanticipated that 300 individuals make their first visits during this period. The sample size was calculated as 169 individuals, with a 95% confidence level and a 5% margin of error. The data of the study were collected via face-to-face survey method after obtaining verbal and written consents. A four-part data collection form was used in the survey. The first part of the form consisted of 20 questions about sociodemographic characteristics and reasons to start and keep smoking. The second part included the Modified Fagerstrom Test for nicotine addiction, consisting of 6 questions. The Turkish validity and reliability of the Modified Fagerstrom Test was made by Uysal et al. [14]. The Modified Fagerstrom Test consists of six questions, each with a different score. Based on the total score obtained from the evaluation of this test, nicotine dependence is graded into three groups: low (0–3 points), moderate (4–6 points), and high (≥ 7 points) [15].The third part of the form included the Burnout Measure-Short Version (BMS) consisting of 10 questions, and the fourth part included the Couple Burnout Measure-Short Version (CBMS) consisting of 10 questions. The 10 items selected for the Burnout Measure short form were determined based on the contextual basis of the 21-item burnout scale, which assesses individuals’ physical, emotional, and mental fatigue levels. The scale is answered on a seven-point scale (1 for Never and 7 for Always) to measure individuals’ occupational burnout levels, based on interviewees’ statements The internal consistency coefficients of the scale, calculated using data from different ethnic backgrounds, professions, and student groups, were observed to range from 0.85 to 0.92. The Cronbach alpha internal consistency coefficient for the Burnout Measure-Short Form was found to be 0.91, item-total test correlations ranged from 0.44 to 0.77, and the correlation coefficient obtained with a four-week test-retest interval was *r* = 0.88 (*p* < 0.01) in the Turkish adaptation study [15, 16].

The short form of the Couple Burnout Measure is answered by interviewers on a seven-point scale (1 for Never and 7 for Always) to measure the level of burnout related to marriage and relationships of people who are married, dating, engaged, or in all types of relationships that can be described as couples. The internal consistency coefficients of the scale, calculated using data obtained from married individuals, were found to be 0.94 for married women and 0.95 for married men. The Cronbach alpha internal consistency coefficient of the Couple Burnout Measure was calculated as 0.91, while the item-total test correlations varied between 0.41 and 0.76, and the test-retest correlation coefficient obtained at a four-week interval was found to be *r* = 0.90 (*p* < 0.01) in the Turkish adaptation study. In the original validation studies, the Cronbach alpha values of BMS and CBMS were both 0.85 [15, 16].

### Statistical analysis

The statistical analysis was conducted using IBM SPSS Statistics for Windows, Version 23 [IBM Corp., Armonk, NY, USA]. Descriptive statistics were presented as mean ± standard deviation for continuous variables and as frequencies and percentages for categorical variables. Group comparisons were performed using the independent samples t-test or one-way ANOVA, and non-parametric alternatives (Mann–Whitney U and Kruskal–Wallis tests) were applied when assumptions were not met. Relationships between categorical variables were examined using the chi-square test, and correlations between continuous variables were assessed using Pearson’s or Spearman’s correlation analyses. A p-value of < 0.05 was considered statistically significant.

## Results

A total of 151 people (58 female and 93 male) were included in the study. The mean age of the participants was 41.17 ± 11.59. 29.1% (44) of the participants were single, 60.3% (91) were married, 10.6% (16) were divorced. 36.4% (55) did not have any child. 19.2% (29) of them stated that they were living alone, 64.9% (98) of them were living with their spouse or children. 84.8% (128) of them were working full time, 12.6% (19) part time and 2.6% (4) of them were not working. The sociodemographic data of the survey participants are given in Table 1.

**Table 1:** Sociodemographic data of the participants

Age	Mean ± SD	41.17 ± 11.59
Gender	Number [*n*]	Percentage [%]
Female	58	38.4
Male	93	61.6
Marital Status	Number [n]	Percentage [%]
Married	91	60.3
Single	44	29.1
Divorced	16	10.6
Number of Children	Number [n]	Percentage [%]
No Child	55	36.4
1 Child	29	19.2
2 Children	39	25.8
3 Children	21	13.9
More Than 3 Children	7	4.6
Who Are You Living With?	Number [n]	Percentage [%]
Alone	29	19.2
Mother/Father	16	10.6
Spouse/Children	98	64.9
Other	8	5.3
Education Level	Number [n]	Percentage [%]
Illiterate	1	0.7
Primary School	17	11.3
Middle School	9	6
High School	49	32.5
Associate’s Degree	10	6.6
Bachelor’s Degree	55	36.4
Master’s Degree	8	5.3
Doctorate	2	1.3
Working Status	Number [n]	Percentage [%]
Full-time	128	84.8
Part-time	19	12.6
Unemployed	4	2.6
Occupation	Number [n]	Percentage [%]
Tradesman	32	21.2
Worker	61	40.4
Officer	58	38.4
Total Household Income	Number [n]	Percentage [%]
5.000–10.000 TL	15	9.9
10.000–15.000 TL	24	15.9
15.000–20.000 TL	27	17.9
20.000–25.000 TL	19	12.6
25.000–30.000 TL	24	15.9
More than 30.000 TL	42	27.8

In the table, data containing more than one group are expressed as numbers and percentages. Data showing a parametric distribution without a group are expressed as “mean±standard deviation”, while data showing a nonparametric distribution are given as “median [lowest value-highest value]”

While 62.9% (95) of the participants were satisfied with their profession, 6.6% (10) were not. 19.9% ​​(30) of the working ones were working in shifts. 60.3% (91) of the participants had no illness, while 10.6% (16) had a psychiatric illness. All but 4 (2.64%) of the 60 (39.7%) individuals with chronic diseases were using medication. 62.3% (94) of the participants stated that they had tried to quit smoking before, and the mean duration of smoking was 20 years. In addition, 49.7% (75) of them were also drinking alcohol. The participants stated that they started smoking mostly because of peer pressure (54.3%), while the main reasons for smoking were because it was enjoyable (34.4%) and because they were addicted (34.4%).

According to the Fagerstrom test results applied to the participants in the study, 45.7% (69) were moderately, 40.4% (61) were highly, and 13.9% (21) were lowly addicted. According to the BMS results, 47% (71) showed low burnout level, while 5.3% (8) should seek professional help as soon as possible. When the CBMS results were evaluated, 55% (83) showed low burnout level, while 2% (3) should seek professional help as soon as possible.

The burnout measure showed a strong positive correlation with the couple burnout measure, and subgroup chi-square analyses were also significant.

All sociodemographic characteristics were analysed whetther there was a relationship between burnout. Number of children, job satisfaction, shift work status and presence of illness changed the burnout measure score at a statistically significant level. When the total burnout measure results were compared between the groups, they were found to be significantly higher in shift workers than in those who did not work shifts (*p* = 0.038). When the burnout measure results were compared according to the number of children, the burnout level was found to be significantly higher in those with no children compared to those with one child (*p* = 0.024) and in those with two children compared to those with one child (*p* = 0.021). No significant difference was found among individuals with other numbers of children. When we evaluated it in terms of job satisfaction, the highest burnout score was seen in those who were dissatisfied and the lowest score was seen in those who were satisfied. When the groups were compared pairwise, the burnout level was found to be significantly higher in those who were dissatisfied with their jobs than in those who were satisfied (*p* = 0.035). At the same time, the burnout level of those who were undecided was found to be significantly higher than those who were satisfied (*p* < 0.001). When the illness status was compared among the participants, the burnout level was found to be significantly higher in those with psychiatric ilness than in those without any illness (*p* = 0.005).

When the couple burnout measure and sociodemographic data were compared, gender and job satisfaction changed the couple burnout measure score at a statistically significant level. When the total results of the couple burnout measure were compared according to gender, the couple burnout measure results were found to be significantly higher in women than in men (*p* = 0.001).

When we evaluated the couple burnout measure results in terms of job satisfaction, the highest couple burnout was seen in those who were dissatisfied and the lowest couple burnout was seen in those who were satisfied. When the groups were compared pairwise, the couple burnout measure level of those who were dissatisfied with their jobs was found to be significantly higher than those who were satisfied (*p* = 0.031). At the same time, couple burnout was found to be significantly higher in those who were undecided than in those who were satisfied (*p* = 0.007).

When Fagerstrom, BMS, CBMS scores were compared with alcohol use and smoking cessation attempts, no significant results were found.

In smoking cessation, it is important to determine the reasons for starting and continuing smoking and to intervene accordingly. When the reasons to start smoking and to keep smoking were compared with the BMS and CBMS results, no significant result was found. (See Table 2)

**Table 2:** Evaluation of burnout and coupe burnout measures according to the reasons to start smoking and keep smoking

Reason to Start Smoking	Burnout Measure					Chi-Square pi value
	< 2.4	2.5–3.4	3.5–4.4	4.5–5.4	> 5.5	
Peer pressure	32	30	12	2	6	0.102
Problems related to work	3	4	2	0	1	0.701
Spouse, girlfriend/boyfriend problems	9	3	1	2	0	0.177
Having smokers in family	8	5	4	1	1	0.896
To avoid being excluded from social circles	18	6	1	3	1	0.055
To appear older	7	3	0	0	0	0.45
Imitation of celebrities	2	0	0	1	1	0.057
Because it gives pleasure	7	5	3	1	2	0.762
Other reasons	1	2	1	0	0	0.785
Reason to Keep Smoking						
It gives pleasure	26	10	8	4	4	0.143
Because I’m addicted	18	19	11	3	1	0.06
It makes me forget/ease my troubles	13	12	4	1	0	0.486
Habitual	33	30	12	3	5	0.304
Psychologically relaxing	12	12	4	1	3	0.559
Other reasons	0	0	0	0	0	1.000

**Reason to Start Smoking**	**Couple Burnout Measure**					**Chi-Square** ***pi*** **value**
	**< 2.4**	**2.5–3.4**	**3.5–4.4**	**4.5–5.4**	**> 5.5**	
Peer pressure	43	14	4	2	3	0.595
Problems related to work	5	3	0	0	0	0.743
Spouse, girlfriend/boyfriend problems	9	3	1	0	0	0.917
Having smokers in family	12	1	2	2	0	0.073
To avoid being excluded from social circles	17	4	1	0	0	0.708
To appear older	6	2	0	0	0	0.896
Imitation of celebrities	2	0	1	0	0	0.285
Because it gives pleasure	11	4	2	0	1	0.599
Other reasons	1	2	0	0	0	0.446
Reason to Keep Smoking						
It gives pleasure	26	9	3	3	2	0.301
Because I’m addicted	25	11	4	2	0	0.31
It makes me forget/ease my troubles	17	7	1	0	0	0.648
Habitual	45	15	4	3	1	0.87
Psychologically relaxing	20	6	1	2	1	0.732
Other reasons	0	0	0	0	0	1.000

## Discussion

Smoking is a serious public health problem today and affects human life. According to the WHO reports, the number of people dying from smoking in developing and underdeveloped countries is expected to increase exponentially [17]. Reasons for smoking may include unhappiness and dissatisfaction, which negatively affect emotional state [18]. Although these reasons play an important role in continuation of smoking, some people may decide to quit smoking to get rid of the effects of all these negative emotions. When we searched the literature, we came across studies comparing depression and anxiety levels in people who decided to quit smoking, with scales. However, we could not find any study measuring burnout, which is a more advanced state of emotional depression. In addition, when deciding to quit smoking, the relationships of a person with the people he/she lives with also affect the quitting process positively or negatively. Therefore, in our study, we aimed to define the burnout states of both occupational and spouse-related individuals who applied to the smoking cessation clinic in order to better understand the emotional aspects of the individuals who decided to quit smoking and to determine the smoking cessation method more accurately. In our study, it was found that the number of children, job satisfaction, shift work status and presence of illness significantly affected the burnout measure score, while gender and job satisfaction significantly affected the couple burnout measure score. When we looked at the total scores, BM and CBM scores were found to be correlated with each other.

In our study, 61.6% of those who decided to quit smoking were male and 38.4% were female. Similar to our study, many other studies have found that the number of male patients applying to the smoking cessation clinics is significantly higher [19–21]. This may be due to the traditional cultural structure of societies, socioeconomic conditions, and the fact that men have more awareness and the opportunity to apply to smoking cessation clinics. It can also be explained by the fact that the prevalence of smoking is higher in men in our country and in the world [22].

In our study, the reason for initiation of smoking was the peer pressure with 54.3%. In the study conducted by Mayda et al. on students in 2010 and by Sahin et al. on people who applied to a smoking cessation center in 2012, the most common reason for starting smoking was imitation [23, 24].

The most common reasons for smoking were addiction and pleasure (34.4%). In the study conducted by Firat et al. in 2019 with the patients who applied to the smoking cessation clinic, the most common reasons for smoking were determined to be stress and the desire to smoke after meals [18]. Although people choose to smoke as a way to escape and relax in temporary negative emotional states, they still continue to smoke just because it gives them pleasure even after that negative situation ceases to exist. Community-based training can be organized to help people to cope with temporary adversities more comfortably and effectively, or they can be provided with the support of a psychologist more easily. In this regard, it can be expected that Healthy Living Centers, which have been operational in recent years and have psychologists on site, will play a more active role.

In addition, the Fagerstrom scale scores of the participants whose reason to start smoking was due to peer pressure were found to be significantly higher. Also, those who chose addiction as the reason for smoking also had a significantly higher Fagerstrom scale score. In a study conducted in Ankara in 2002, when students were asked whether their friends smoked or not, 38.4% of them said they smoked and 55.2% said they did not smoke. The relationship between the smoking status of an adolescent’s friends, especially their close friends, and that adolescent’s smoking status was found to be statistically significant [25]. It has been observed that the peer pressure and starting smoking at an early age have an effect on the addiction of young people and that they continue to smoke for this reason [26]. The findings in this research support the results of our study.

In our study, when the total burnout measure results were compared between the groups, they were found to be significantly higher in shift workers than in those who did not work shifts. In a study conducted by Ozturk et al. in 2014 on 403 nurses, the night workers were found to have higher depersonalization and emotional exhaustion scores [27]. In a study conducted in Brazil it was found that professionals working the night shift, having low social support, being dissatisfied with sleep, having children, not having a religion, having worked for a short period in the institution, and being a nursing technician or aid were significantly more likely to experience high levels of the syndrome [28].

When the illness status of the participants in our study was compared, the burnout level was found to be significantly higher in those with psychiatric illness than in those without any illness, as expected. In the study conducted by Arpacioglu et al. in 2021, it was stated that as burnout level increases in healthcare workers, depression also increases, meaning there is a positive correlation between them [29]. In the research conducted by Ozulku in Istanbul in 2021, as the level of burnout increased in healthcare workers, the level of depression also increased [30]. The results of these studies also support our study.

In our study, the couple burnout measure results were found to be significantly higher in women than in men. In a study conducted by Metin et al. in 2005, it was stated that women’s excessive household responsibilities in addition to their work life and not being helped at home increased their level of burnout [31]. Due to the correlation between burnout and couple burnout, it was considered an expected result that women with increased burnout levels would also have higher couple burnout levels.

Studies have shown that occupational burnout and couple burnout develop parallel to each other and affect each other. It has been observed that problems in people’s relationships affect burnout at work, and problems at work affect burnout in relationships [9, 12]. In our study, the burnout measure showed a strong positive correlation with the couple burnout measure.

Strengths of the study: The study was conducted using validated instruments and both occupational and relational burnout were explored.

Limitations of the study: The sample size was calculated as 169 individuals, But we have reached 151 individuals. Limitation of the recruitment to six months and lower number of patients at than anticipated are the major causes.

The other limitation is that, the study was conducted at two centers which are in the same region. The sociodemographic characteristics of the patients are similar but these centers can not represent the entire population. Because patient characteristics can change in different centers.

## Conclusion

In people who decide to quit smoking, the underlying burnout should definitely be taken into consideration. In particular, people should talk to their spouses and tell them that spousal support is very important during this period and that they should be more understanding towards them. Psychologist and, if necessary, psychiatrist support should be provided in the treatment.

However, in order to help these individuals more effectively, more studies are needed to be conducted to evaluate the factors that lead to burnout and couple burnout.

## Supplementary Information

Below is the link to the electronic supplementary material.

Supplementary Material 1.

Supplementary Material 2.

## Data Availability

The data that support the findings of this study are openly available in [Hacettepe University Open Access System] at [https://openaccess.hacettepe.edu.tr/xmlui/handle/11655/34977].
