# Supplementary material for: Evaluation of burnout level in working people who decided to quit smoking
Source: Ir J Med Sci. 2026 Mar 31;195(3):1423–30. doi: 10.1007/s11845-026-04329-8 (PMC13342331; doi:10.1007/s11845-026-04329-8)
Supplement: Supplementary file 1 — Supplementary Material 1. [file 11845_2026_4329_MOESM1_ESM.pdf]

Authorship Form

Irish Journal of Medical Science

Springer

Manuscript ID Number: ....

Article Title: (first few words) Evaluation of Burnout Level

First Author: Asena Merve Oflaz

E-mail: mrvoflazz@gmail.com

AUTHORSHIP

I, the undersigned author(s), certify that:

- I have seen and approved the final version of the manuscript, and all subsequent versions;
- I have made substantial contributions to conception and design, or acquisition of data, or analysis and interpretation of data;
- I have drafted the article or revised it critically for important intellectual content.
- I agree to be accountable for all aspects of the work in ensuring that questions related to the accuracy or integrity of any part of the work are appropriately investigated and resolved.

I accept public responsibility for it, and believe it represents valid work. As an author of this article, I certify that none of the material in the manuscript has been previously published, nor is included in any other manuscript. I certify that this manuscript is not under consideration for publication elsewhere, nor has it been submitted or accepted in another publication in any form. The rights or interest in the manuscript have not been assigned to any third party.

Moreover, should the editor of *Irish Journal of Medical Science* request the data upon which the manuscript is based, I shall produce it. I also certify that I have read and complied with the copyright information, as found on the journal home page website.

After submission of this agreement signed by all authors, changes of authorship or in the order of the authors listed will not be accepted by Springer.

☐ Author's signature	☐ Author's signature
Asena Merve Oflaz & 05.09.25<br>Printed name & date	Duygu Ayhan Başer, 08.09.25<br>Printed name & date
☐ Author's signature	☐ Author's signature
Hilal Aksoy & 05.09.25<br>Printed name & date	Printed name & date
☐ Author's signature	☐ Author's signature
İzzet Fidancı & 05.09.25<br>Printed name & date	Printed name & date
☐ Author's signature	☐ Author's signature
İsmail Arslan & 08.09.25<br>Printed name & date	Printed name & date

Completed forms can be scanned and included as a pdf file during the online submission process as a supplemental file not for review, or submitted by fax to the editorial office: +91 44 42197763

Scanned with CamScanner
